# Impact of low-dose sufentanil on the effective sedative dose of ciprofol for BIS-guided induction in elderly patients: an up-and-down sequential allocation trial

**DOI:** 10.3389/fmed.2025.1715148

**Published:** 2026-01-29

**Authors:** Qing Han, Chun-ming Hu, Jun-li Zheng, Xiao-dong Huang, Pei Chen, Wei-long Wang, Jin Zhou, Zhen-feng Zhou

**Affiliations:** 1 Department of Anesthesiology, The First People's Hospital of Linping District, Hangzhou, Zhejiang, China; 2 The Fourth School of Clinical Medicine, Zhejiang Chinese Medical University, Hangzhou, Zhejiang, China; 3 Department of Anesthesiology, Hangzhou Children's Hospital, Hangzhou, Zhejiang, China; 4 Department of Anesthesiology, Hangzhou Women's Hospital (Hangzhou Maternity and Child Health Care Hospital, Hangzhou First People's Hospital Qianjiang New City Campus, Zhejiang Chinese Medical University), Hangzhou, China

**Keywords:** ciprofol, sufentanil, elderly, induction of anesthesia, effective dose

## Abstract

**Objective:**

This study aimed to evaluate the effect of a single low dose of sufentanil on the effective dose of ciprofol required to achieve a bispectral index (BIS) <60 during anesthesia induction in elderly patients.

**Methods:**

A total of 48 elderly patients were randomly assigned to either the sufentanil plus ciprofol group (S + C group) or the ciprofol alone group (C group). A sufentanil dose of 0.1 μg/kg was administered to the S + C group (diluted to 5 ml), while the C group was administered 5 ml of normal saline. Five minutes later, the initial administration for both groups was 0.3 mg/kg ciprofol. Subsequent doses were adjusted in increments or decrements of 0.05 mg/kg according to the response of the preceding patient within the same treatment group, following two independent, arm-specific up-and-down sequences conducted in parallel. Successful sedation was defined as achieving a BIS score of < 60 within 5 min following ciprofol administration. ciprofol's effective doses (ED50/ED95) were derived through probit regression.

**Results:**

A total of 23 patients were enrolled in the S + C group and 25 in the C group. The estimated ED50 of ciprofol was 0.075 mg/kg (95% CI: 0.024–0.123 mg/kg) in the S + C group and 0.267 mg/kg (95% CI: 0.159–0.361 mg/kg) in the C group. The estimated ED95 values were 0.246 mg/kg (95% CI: 0.141–14.566 mg/kg) and 0.439 mg/kg (95% CI: 0.340–67.768 mg/kg), respectively. The Pearson goodness-of-fit test of group S + C and group C were *P* = 0.965 and *P* = 0.615, respectively. The incidence of adverse events, including hypotension (39% vs. 64%) and respiratory depression (17% vs. 16%), did not differ significantly between S + C group and C group.

**Conclusion:**

Under BIS monitoring, the estimated ED50 and ED95 of ciprofol for induction in elderly patients were 0.267 and 0.439 mg/kg, respectively, without sufentanil, and 0.075 and 0.246 mg/kg with 0.1 μg/kg sufentanil. The addition of a low dose of sufentanil reduced the ciprofol requirement for BIS-targeted induction by about 44%−72% without increasing the incidence of hypotension or respiratory depression. This regimen provides an effective and well-tolerated strategy for anesthesia in elderly patients, particularly in day surgery and outpatient settings.

**Clinical trial registration:**

http://www.chictr.org.cn, identifier: ChiCTR2400090926.

## Key points

1. Under BIS monitoring, the estimated ED50 and ED95 of ciprofol for induction in elderly patients were 0.267 and 0.439 mg/kg, respectively, without sufentanil, and 0.075 mg/kg and 0.246 mg/kg with 0.1 μg/kg sufentanil.
2. Compared with ciprofol alone, sufentanil reduced the ciprofol requirement for BIS-targeted induction by about 44%−72% in elderly patients.
3. Low-dose sufentanil did not increase the incidence of hypotension or respiratory depression.

## 1 Introduction

Ciprofol is a novel Class I intravenous anesthetic that functions similarly to propofol. It acts as a γ-aminobutyric acid type A (GABA_A_) receptor agonist, enhancing chloride ion influx across neuronal membranes to induce hyperpolarization and central nervous system inhibition. Compared with propofol, ciprofol exhibits 4–5 times greater potency and provides rapid anesthetic induction, stable hemodynamics, reduced postoperative visitation in elderly patients, limited respiratory depression, faster recovery, and an overall favorable safety profile (1–5).

Elderly patients typically require lower doses of anesthetic agents during general anesthesia due to age-related changes in drug pharmacokinetics and pharmacodynamics (6–9). Minimizing anesthetic dosage in this population is essential to reduce potential complications. However, clinical data on the use of ciprofol in elderly patients remain limited. Current prescribing information advises caution in individuals aged ≥65 years and recommends initiating treatment with a reduced dose.

Existing studies have compared ciprofol use between elderly and non-elderly patients (10). One study demonstrated comparable pharmacokinetics and pharmacodynamics at 0.3 mg/kg in elderly and 0.4 mg/kg in younger patients. Another investigation estimated ED50 of ciprofol in older adults to be between 0.263 and 0.267 mg/kg (11). However, these studies did not assess the influence of opioid coadministration on ciprofol's effective dose.

The synergistic interaction between sedatives and opioids in modulating sedation depth remains controversial. Our previous findings indicated that 1 μg/kg fentanyl reduced the induction dose of remimazolam by approximately 30% in elderly patients while maintaining stable hemodynamics and respiratory function (12). Similar studies (13, 14) have reported that coadministration of analgesics may lower the required sedative dose. In contrast, other research (15, 16) suggests that opioids may not significantly influence the sedative depth achieved with agents such as propofol.

The present study aimed to investigate the effect of a single low dose of sufentanil on the effective dose of ciprofol required to achieve a BIS < 60 during general anesthesia induction in elderly patients, particularly for day surgery or outpatient settings.

## 2 Materials and methods

### 2.1 Study design

Ethical approval for this study (IRB: 2024 Study No. 196) was provided by the Ethics Committee of the First People's Hospital of Lin-ping District, Hangzhou (Chairman Prof Ming-hua Xie) on October 12, 2024. Clinical trial registration number: ChiCTR2400090926. Written consent forms were completed by each participant before study participation. A total of 55 elderly patients scheduled for elective surgery under general anesthesia were enrolled between October 28, 2024, and January 30, 2025.

### 2.2 Inclusion and exclusion criteria

Inclusion criteria were as follows: (1) age between 65 and 85 years; (2) scheduled for surgery under general anesthesia; (3) American Society of Anesthesiologists (ASA) physical status I–II; (4) body mass index (BMI) between 18 and 25 kg/m^2^; and (5) voluntary participation with signed informed consent.

Exclusion criteria included: (1) known allergy to ciprofol (e.g., soybean allergy) or contraindications to its use; (2) preoperative cognitive impairment or chronic pain requiring long-term use of analgesics, psychotropics, NSAIDs, or sedatives; (3) anticipated difficult airway or history of abnormal anesthesia responses; (4) presence of malignancies or severe cardiovascular/cerebrovascular disease; (5) use of sedatives, antiemetics, antipruritics, monoamine oxidase inhibitors, or antidepressants within 24 h prior to surgery; (6) participation in other drug trials; and (7) requirement for emergency surgery.

### 2.3 Randomization and blinding

Randomization was performed using a computer-generated sequence and implemented via sequentially numbered, opaque, sealed envelopes. An independent anesthesia nurse who was not involved in any anesthesia care or data collection would open the sealed envelopes containing group allocations and prepare medications according to the group allocations. Patients were randomly assigned to either the sufentanil plus ciprofol group (S + C group) or the ciprofol-alone group (C group).

An independent anesthesiologist was responsible for anesthesia management and data recording, while a separate investigator performed statistical analyses. The anesthesiologist who knows nothing about the details of the medication recorded each patient's reaction and reports it to the independent anesthesia nurse, then this nurse could prepare the medication for the next case using 20-ml syringes. The anesthesiologists, surgeons, patients, and data analysts were all blinded to group allocation.

### 2.4 Anesthesia procedure

Upon arrival in the operating room, standard intraoperative monitoring was initiated, including heart rate (HR), non-invasive blood pressure (NIBP), peripheral oxygen saturation (SpO_2_), and temperature. Invasive monitoring was applied if clinically indicated. All patients underwent intravenous cannulation with an 18–20 G catheter and received fluid loading with lactated Ringer's solution at 3 ml/kg. Preoxygenation was performed prior to anesthesia induction.

### 2.5 Administration of ciprofol

Patients in the S + C group received sufentanil 0.1 μg/kg diluted to 5 ml, while those in the C group received 5 ml of normal saline. Five minutes later, both groups received an initial dose of ciprofol at 0.3 mg/kg. Subsequent doses were adjusted in increments or decrements of 0.05 mg/kg according to the response of the preceding patient within the same treatment group, following two independent, arm-specific up-and-down sequences conducted in parallel. Once sedation was successfully achieved at a 0.05 mg/kg dose, further dose adjustments were halved to increase precision.

Successful sedation was defined as achieving a BIS score < 60 within 5 min after ciprofol administration (17). Sedation failure was defined as a BIS score remaining ≥60 at 5 min after administration, in such cases, an additional 0.05 mg/kg of ciprofol was administered every 3 min until adequate sedation was achieved. Once adequate sedation was achieved, additional sufentanil (0.3 μg/kg) and rocuronium (0.5 mg/kg) were administered to facilitate laryngeal mask or endotracheal tube insertion. BIS was maintained between 40 and 60 during the surgical procedure.

Injection pain and adverse events (e.g., hypotension, bradycardia, respiratory depression, muscle tremors, postoperative nausea and vomiting) were recorded, and management of complications were detailed in Supplementary Appendix 1.

### 2.6 Primary outcome

The primary outcome was the rate of successful sedation, defined by achieving a BIS value < 60 within the first 5 min following ciprofol administration.

### 2.7 Sample size

The sample size was determined according to recommendations indicating that 20–50 participants are sufficient to obtain a stable estimate of the ED50 using the Dixon up-and-down method (18). Furthermore, according to the Dixon sequential design, at least six crossovers (i.e., transitions from failure to success or vice versa) are required to estimate the dose–response relationship (17).

Therefore, if at least six crossovers occurred among the initial 25 participants per group, the trial could be concluded. If six crossovers were not achieved, an additional 25 participants per group would be enrolled. Accordingly, 25 subjects were initially included in each group.

### 2.8 Statistical analysis

Data were analyzed using SPSS 25.0. Continuous variables with normal distribution were reported as mean ± standard deviation and compared using unpaired *t*-tests. Non-normally distributed data were presented as median (interquartile range) and analyzed using the Mann–Whitney *U*-test. Categorical variables were expressed as counts and percentages and compared using the chi-square or Fisher's exact test, as appropriate. ED50 and ED95 of ciprofol were estimated using Probit regression. A two-sided *P*-value < 0.05 was considered statistically significant.

## 3 Results

### 3.1 Patient enrollment and baseline characteristics

**Figure 1:**
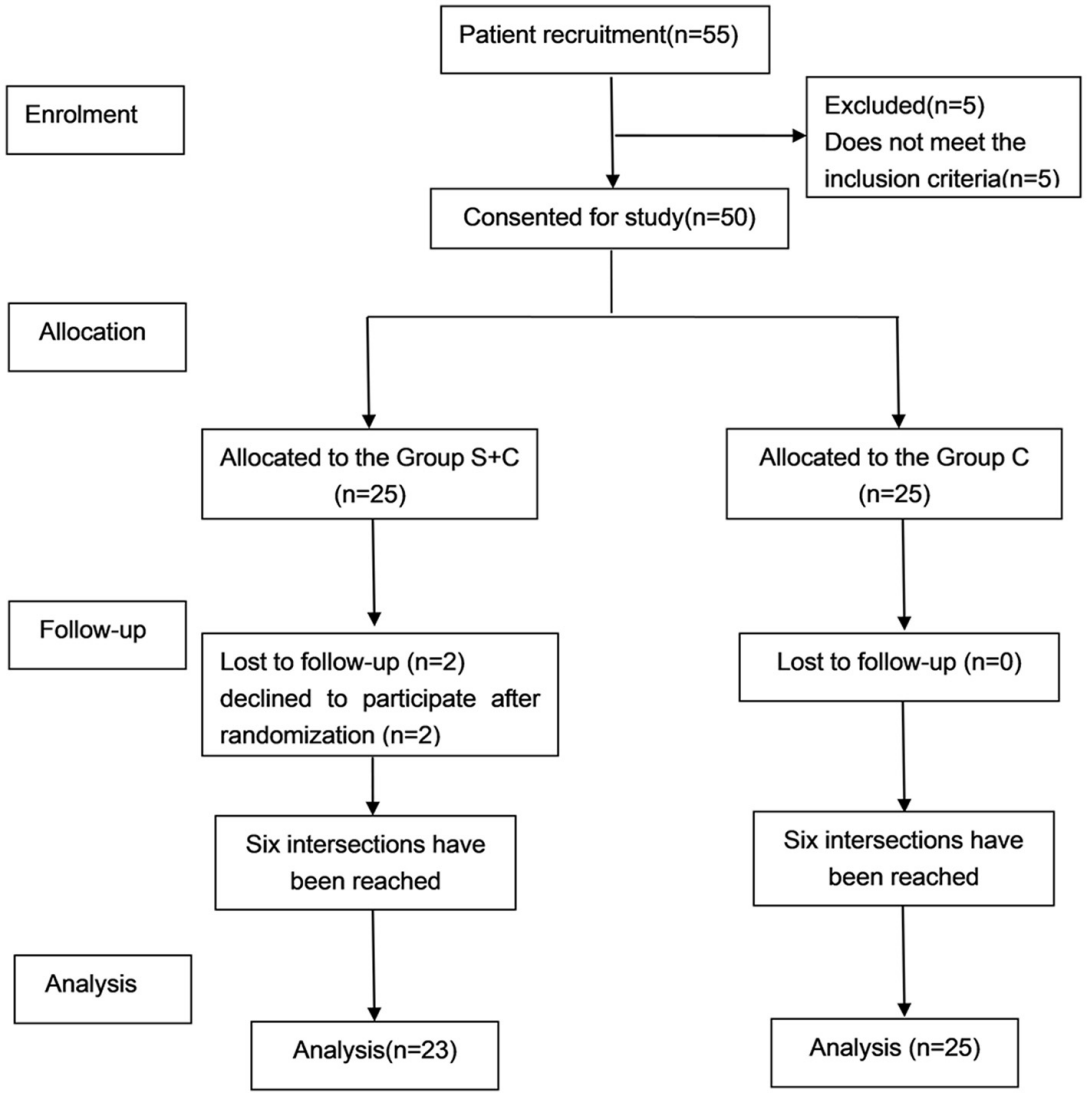
Flow diagram.

**Figure 2:**
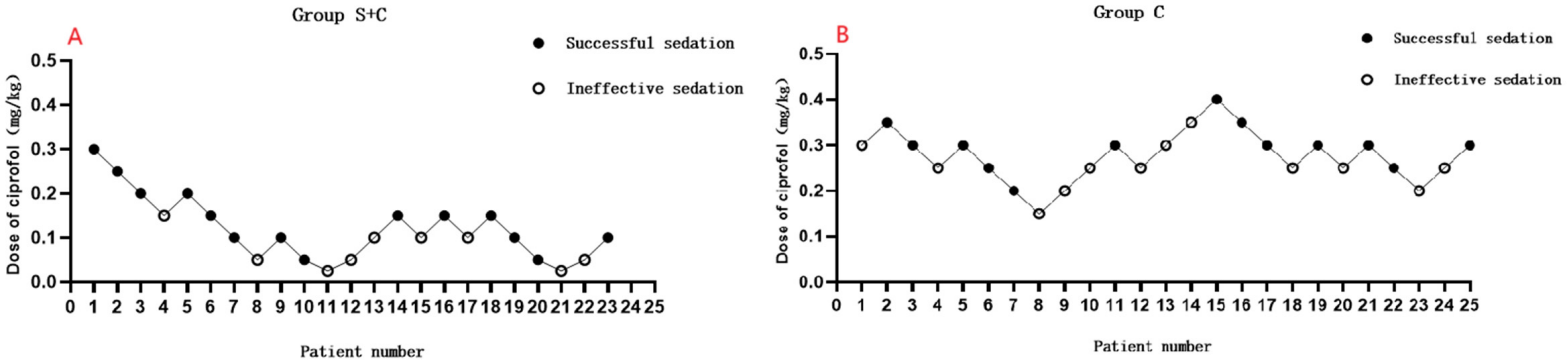
**(A)** Flow chart of sequential trial in sufentanil + ciprofol group; **(B)** Flow chart of sequential trial in ciprofol group.

Initially, 55 patients were recruited for the study, with five excluded for not meeting inclusion criteria. Two declined to participate after randomization in the S + C group. Subsequently, the final analysis was conducted on a cohort of 48 patients, as outlined in Figure 1. A total of six crossover points were obtained in each group (Figure 2). Baseline characteristics including age, BMI, sex distribution, ASA physical status, comorbidities, surgical profiles, and baseline BIS were comparable between the two groups (*P* > 0.05), as shown in Table 1.

**Table 1:** Baseline characteristics of participants.

**Baseline characteristics**	**Sufentanil + ciprofol group (*N* = 23)**	**Ciprofol group (*N* = 25)**
Age, years	68.52 ± 2.23	67.96 ± 2.65
BMI, kg/m^2^	20.72 ± 1.02	21.04 ± 0.87
Female, no. (%)	10 (43.5%)	10 (40%)
ASA, I/II/III, no.	0/23/0	0/25/0
Smoking, no. (%)	5 (21.7%)	7 (28%)
Baseline BIS	94 (89, 98)	97 (91, 98)
**Coexistent disease, no. (%)**		
Hypertension	39.2%	48%
Diabetes	0 (0%)	1 (4%)
COPD	1 (4.3%)	0 (0%)
**Type of surgery, no. (%)**		
Abdominal surgery	12 (52.1%)	10 (40%)
Department of stomatology	1 (4.3%)	0 (0%)
Gynecology	1 (4.3%)	7 (28%)
Urology	5 (21.7%)	3 (12%)
Orthopedics	3 (13%)	2 (8%)
Thoracic surgery	1 (4.3%)	1 (4%)
Vascular surgery	0 (0%)	1 (4%)
Breast surgery	0 (0%)	1 (4%)

BMI, body mass index; ASA, American Society of Anesthesiologists; COPD, chronic obstructive pulmonary disease.

### 3.2 Effective sedative dose of ciprofol (ED50 and ED95) with and without low-dose sufentanil

**Figure 3:**
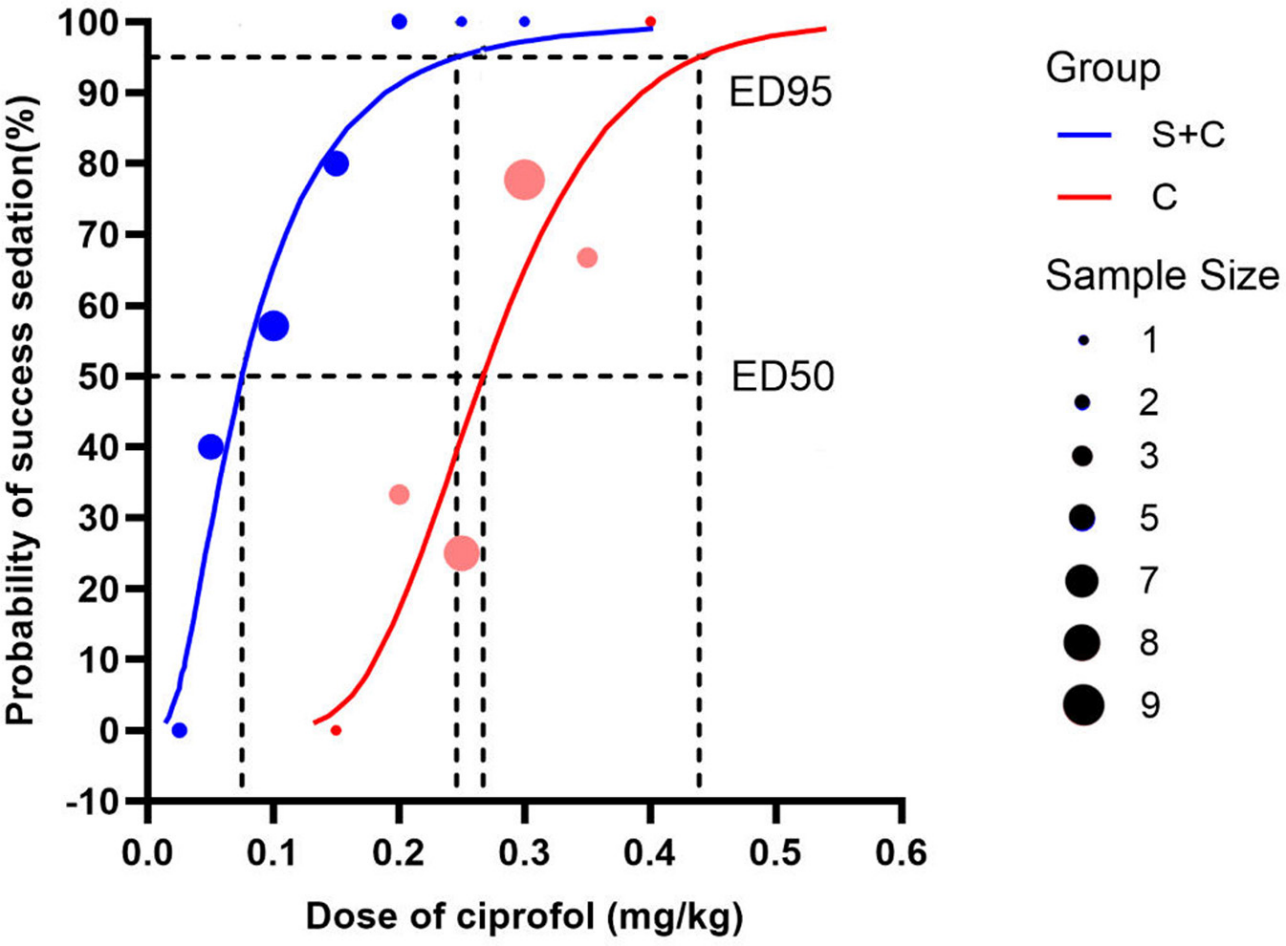
Under BIS monitoring, the dose-response curve of intravenous injection of ciprofol for anesthesia and sedation was drawn according to the effective reaction probability (1%−100%) and the corresponding dose of initial injection obtained by probit regression analysis. The ED50 and ED95 of ciprofol in sufentanil + ciprofol group and ciprofol group were calculated by probit regression. The dashed line indicates the ED50 value and the solid line indicates the ED95 value.

The up-and-down sequential allocation flowcharts for both groups were presented in Figure 2. Probit regression analysis estimated the ED50 of ciprofol in the S + C group to be 0.075 mg/kg (95% CI: 0.024–0.123 mg/kg), and the ED95 to be 0.246 mg/kg (95% CI: 0.141–14.566 mg/kg), Pearson goodness-of-fit test showed *P* = 0.965. In contrast, the ED50 and ED95 in the ciprofol-alone group were 0.267 mg/kg (95% CI: 0.159–0.361 mg/kg) and 0.439 mg/kg (95% CI: 0.340–67.768 mg/kg), Pearson goodness-of-fit test showed *P* = 0.615. The co-administration of low-dose sufentanil therefore reduced the ciprofol requirement for achieving BIS-targeted induction by approximately 44%−72% (ED50 ratio: 0.075/0.267 = 0.28; 95% CI: [0.112, 0.701]). The dose-response curves for both groups are illustrated in Figure 3.

### 3.3 Adverse events

Adverse events observed during anesthesia induction are summarized in Table 2. In the S + C group, four patients (17%) experienced respiratory depression, and nine patients (39%) developed hypotension. In the ciprofol-alone group, respiratory depression occurred in four patients (16%), hypotension in 16 patients (64%), and muscle tremors in two patients (8%). The risk ratio for hypotension between the two groups was 0.61 (*P* = 0.085), and that for respiratory depression was 1.06 (*P* > 0.999). No cases of bradycardia, injection site pain, or postoperative nausea and vomiting were observed in either group.

**Table 2:** Adverse events.

**Adverse events, *n* (%)**	**Sufentanil + ciprofol group (*N* = 23)**	**Ciprofol group (*N* = 25)**	**RR**
Hypotension	9 (39%)	16 (64%)	0.61
Bradycardia	0 (0%)	0 (0%)	0
Respiratory depression	4 (17%)	4 (16%)	1.06
Postoperative nausea and vomiting (1/2/3)	0/0/0	0/0/0	0
VRS of pain intensity (1/2/3)	0/0/0	0/0/0	0
Muscle tremors	0 (0%)	2 (8%)	0

VRS, verbal rating scale.

## 4 Discussion

Under bispectral index (BIS) monitoring, the estimated ED50 and ED95 of ciprofol for induction in elderly patients were 0.267 and 0.439 mg/kg, respectively, when administered without sufentanil. Co-administration of 0.1 μg/kg sufentanil markedly reduced these doses to 0.075 mg/kg (ED50) and 0.246 mg/kg (ED95), The addition of a low dose of sufentanil reduced the ciprofol requirement for BIS-targeted induction by about 44%−72%. Importantly, this dose-sparing effect was not associated with an increased incidence of hypotension or respiratory depression. This opioid–sedative synergy is more pronounced than the 30% dose-sparing effect we previously observed with 1 μg/kg fentanyl combined with remimazolam (12). Chen et al. (19) eported that combining sufentanil with remimazolam during anesthesia induction in elderly patients significantly reduced the required dose of remimazolam, consistent with our findings.

This enhanced effect may be attributed to the included old patients and the potent analgesic properties of sufentanil, which alleviate pain-induced stress responses and subsequently lower the required dose of sedatives. Ciprofol, a highly lipophilic GABA_A_ receptor agonist with four- to five-fold greater potency than propofol (20), produces rapid cortical suppression that is likely amplified by opioid-induced reductions in arousal pathways. Beyond analgesia, opioids are known to exert sedative properties, with electrocortical activity patterns resembling those observed during sleep or general anesthesia (20). Among opioids, sufentanil stands out for its high receptor affinity, rapid metabolism, and limited cardiovascular effects—attributes that make it especially suitable for anesthesia induction in elderly patients.

Clinically, a Bispectral Index (BIS) value below 60 is widely accepted as indicative of unconsciousness (17). Substantial evidence supports BIS monitoring as a reliable correlate of sedation depth for agents such as propofol (21, 22), establishing its utility in predicting loss of consciousness. Given that ciprofol and propofol share similar mechanisms of action as GABA_A_ receptor agonists, BIS monitoring is presumed equally applicable for assessing anesthetic depth during ciprofol administration. Previous research further confirms a strong correlation between BIS values and ciprofol-induced sedation (23), reinforcing its role in ensuring adequate anesthetic depth and safety. In the present study, BIS monitoring was particularly critical due to the advanced age of the patient cohort (65–85 years), a population at increased risk for intraoperative awareness and postoperative cognitive dysfunction. Consequently, precise titration of sedation depth was essential in this group.

Our findings further indicate that adding sufentanil to ciprofol did not significantly increase respiratory depression, suggesting no substantial elevation in respiratory risk with co-administration. Similarly, our previous studies demonstrated that co-administration of 1 μg/kg fentanyl with remimazolam did not increase the incidence of adverse effects such as hypotension or respiratory depression in either elderly (12) or non-elderly (14) patients. Neither group exhibited postoperative nausea and vomiting, indicating favorable gastrointestinal tolerability-potentially attributable to ciprofol's pharmacological properties, which mirror propofol's established antiemetic effects (24).

Regarding hemodynamic stability, intraoperative hypotension during induction was lower in the combination group (39%) than in the ciprofol-alone group (64%). Previous studies have also shown that (25) sufentanil combined with propofol stabilized intraoperative hemodynamic parameters, reduced perioperative stress and pain, and decreased sedative requirements by approximately 44%. It can be seen from this that the reason for the difference in the incidence of hypotension between the two groups may be related to the reduction in the dosage of ciprofol after the combination of sufentanil.

Muscle tremors occurred in 8% of the ciprofol-alone group but were absent in the combination group, suggesting that sufentanil's potent analgesia and stress-response suppression may mitigate neuromuscular excitability. Local injection pain was not observed in both groups, probably because ciprofol was formulated as an oil-in-water emulsion because of its aqueous insolubility. In addition, the higher hydrophobicity and lower plasma concentration of ciprofol may have led to the reduction in injection pain (26, 27).

### 4.1 Limitations

Several limitations should be acknowledged. First, this study included only relatively healthy elderly patients classified as ASA II. The approach warrants additional evaluation in vulnerable geriatric patients (ASA grade ≥III). Furthermore, individuals exceeding 85 years of age were not enrolled, necessitating additional research into ciprofol's pharmacokinetics in this age group. In addition, BIS-only success may over- or under-estimate clinical unconsciousness, especially with opioids. Finally, the study's sample size was limited, potentially restricting the statistical power to detect differences in adverse event rates between groups. We also observed that the 95% confidence intervals (CIs) for the ED95 values in both groups were relatively wide and overlapping, suggesting that the upper tail of the dose-response curve was not well defined under the up-and-down sequential allocation design. Therefore, the estimated reduction in the ED95 of ciprofol should be interpreted as an approximate rather than a precise value.

## 5 Conclusions

Under BIS monitoring, the estimated ED50 and ED95 of ciprofol for induction in elderly patients were 0.267 and 0.439 mg/kg, respectively, without sufentanil, and 0.075 and 0.246 mg/kg with 0.1 μg/kg sufentanil. The addition of a low dose of sufentanil reduced the ciprofol requirement for BIS-targeted induction by about 44%−72% without increasing the incidence of hypotension or respiratory depression. This regimen provides an effective and well-tolerated strategy for anesthesia in elderly patients, particularly in day surgery and outpatient settings.

## Data Availability

The raw data supporting the conclusions of this article will be made available by the authors, without undue reservation.
