# Supplementary material for: Impact of low-dose sufentanil on the effective sedative dose of ciprofol for BIS-guided induction in elderly patients: an up-and-down sequential allocation trial
Source: Front Med (Lausanne). 2026 Jan 29;12:1715148. doi: 10.3389/fmed.2025.1715148 (PMC12894343; doi:10.3389/fmed.2025.1715148)
Supplement: Supplementary file 1 [file Table_1.DOC]

**S1 Supplemental Appendix 1. Management of Complications.**

During anesthesia, patients may have some adverse reactions, such as hypotension, bradycardia and respiratory depression etc. These conditions need to be identified and symptomatic treatment in time to ensure the safety of patients and the smooth progress of anesthesia.

(1) Hypotension can be defined when the systolic blood pressure is reduced by at least 20% from the baseline systolic blood pressure^[1]^. Once hypotension is found, it can be quickly corrected by 0.9% sodium chloride injection of 200 ml. For hypotension that cannot be corrected by rapid fluid rehydration, appropriate vasoactive drugs are given according to the situation, such as intravenous injection of mehydroxylamine 0.5 mg, which can be interrupted and repeated if necessary. Attention should be paid to the dosage and speed when used to avoid adverse reactions such as arrhythmia.

(2) Bradycardia is another adverse reaction that may occur during anesthesia. When the patient's heart rate is below 50 beats/hour, it is defined as bradycardia^[2]^, and atropine injection 0.5 mg intravenous injection is given.

(3) Respiratory depression^[3]^is respiratory rate <8 breaths/min for >60 seconds and/or SpO₂ <90% and support the lower jaw to keep the airway unobstructed, improve ventilation function, and perform tracheal intubation if necessary.

(4) Pain related to injection is a significant side effect.The intensity of injection pain was evaluated using a 4-point verbal rating scale, encompassing scores ranging from 0 (absence of pain) to 3 (severe pain), and injection pain was identified as the verbal rating scale of 1-3^[4]^. Patients experiencing pain can slow down the speed of drug injection.

(5) Postoperative nausea and vomiting：The severity of PONV is assessed using a Verbal Rating Scale (VRS): 0 indicates no nausea; 1 indicates mild nausea with one episode of vomiting; 2 indicates moderate nausea with less than three episodes of vomiting; and 3 indicates severe nausea with three or more episodes of vomiting^[5]^. Antiemetic drugs can be given for moderate to severe nausea and vomiting.

(6) muscle tremors is a sudden, brief, lightning-like muscle jerk arising from an abnormality of the nervous system, excluding short or prolonged movements caused by the muscle itself such as fasciculation, spasms, or cramps^[6]^.

**References**

[1] Saugel B, Sessler D I. Perioperative Blood Pressure Management[J]. Anesthesiology, 2021, 134(2): 250-261.

[2] Lan H, Liu S, Liao Y, et al. EC50 and EC95 of Remifentanil for Inhibiting Bronchoscopy Responses in Elderly Patients During Fiberoptic Bronchoscopy Under Ciprofol Sedation: An Up-and-Down Sequential Allocation Trial[J]. Drug Design, Development and Therapy, 2024, 18: 6487-6497.

[3] Schüttler J, Eisenried A, Lerch M, et al.. Pharmacokinetics and Pharmacodynamics of Remimazolam (CNS 7056) after Continuous Infusion in Healthy Male Volunteers: Part I. Pharmacokinetics and Clinical Pharmacodynamics[J]. Anesthesiology, 2020, 132(4): 636-651.

[4] Huang X D, Chen J B, Dong X Y, et al. The Impact of Fentanyl on the Effective Dose of Remimazolam-Induced Sedation in Elderly Female Patients: An Up-and-Down Sequential Allocation Trial[J]. Drug Design, Development and Therapy, 2024, Volume 18: 3729-3737.

[5] Zhang H, Zhang M, Hao L, et al. Comparison of the Effects of Ciprofol and Propofol on Postoperative Nausea and Vomiting in Patients Undergoing Outpatient Hysteroscopy[J]. Drug Design, Development and Therapy, 2024, Volume 18: 5701-5707.

[6] Caviness J N. Treatment of Myoclonus[J]. Neurotherapeutics, 2014, 11(1): 188-200.
